# Pott's Puffy Tumor with Intraorbital Abscess

**DOI:** 10.1590/0037-8682-0126-2022

**Published:** 2022-09-30

**Authors:** Elif Gozgec, Hayri Ogul, Korhan Kılıc

**Affiliations:** 1 Ataturk University, Medical Faculty, Department of Radiology, Erzurum, Turkey.; 2 Duzce University, Medical Faculty, Department of Radiology, Duzce, Turkey.; 3 Ataturk University, Faculty of Medicine, Department of Otorhinolaryngology, Erzurum, Turkey.

**FIGURE 1:**
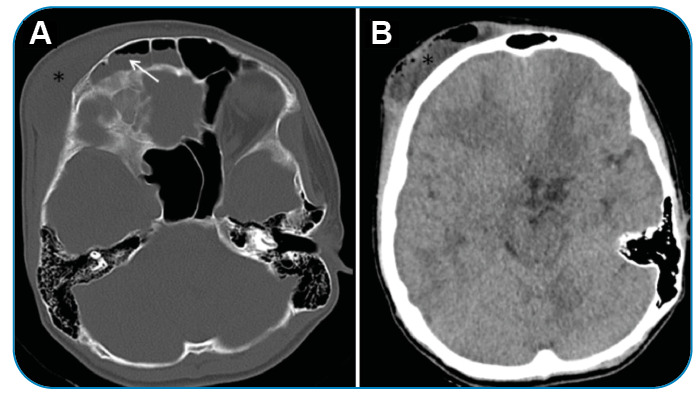
On CT images in the axial section, bone **(A)**, and parenchyma **(B)** window, a lesion (asterix) consistent with a subperiosteal abscess is seen, containing air densities in the right frontal region, and density increases (arrow) in the right frontal sinus.

**FIGURE 2:**
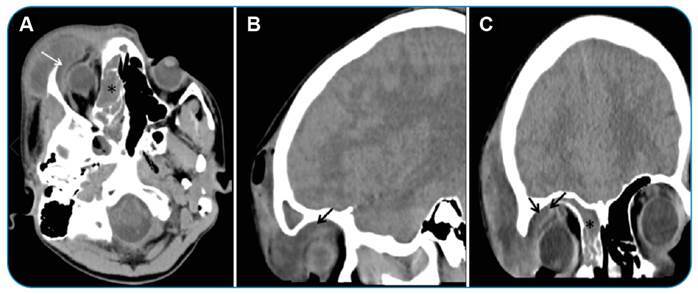
Axial **(A)**, sagittal **(B)**, and coronal **(C)** sectional images of the brain CT show the extension of the subperiosteal abscess into the orbit (arrows) and the density of ethmoid cells on the right side.

A 15-year-old male patient was admitted with complaints of headache, facial pain, nasal obstruction, and swelling of the forehead. He had no history of trauma or surgery. The patient was afebrile and had a soft mass lesion in the right frontal region and edema that spread to the face. The neurologic examination was unremarkable, and cervical lymphadenopathy was not detected. A computed tomography (CT) scan of the brain was performed to determine possible sinusitis complications. Images showed density elevations in the ethmoid cells in the right frontal sinus. The subperiosteal abscess was defined as an area of approximately 5.2 x 4.8 x 3.5 cm, with thick and contrasting walls containing air density and loculated fluid originating from the right frontal sinus (Figure 1). The abscess extended from the superior-lateral wall of the orbit to the intraorbital region (Figure 2). An increase in the density and thickness of the right periorbital area was observed, indicating periorbital cellulitis. Intracranial complications were not observed. The patient was started on intravenous antibiotic therapy. Right unsinectomy and abscess drainage were performed by an otolaryngologist and an ophthalmologist. The patient was discharged without postoperative complications. 

Pott's puffy tumor is a life-threatening subperiosteal abscess that develops behind the frontal bone^1^. The most common orbital complications, occurring in 29% of cases, are preseptal and periorbital cellulitis; however, intraorbital abscesses are very rare^2^. Rapid diagnosis and early intervention are important to reduce the morbidity and mortality of the disease. Imaging methods, especially CT with contrast, is crucial for diagnosis^1^ ^,^ ^2^.
